# Budget monitoring, accountability practices and their influence on the efficiency of county health systems in Kenya

**DOI:** 10.1371/journal.pgph.0001908

**Published:** 2023-11-16

**Authors:** Anita Musiega, Lizah Nyawira, Benjamin Tsofa, Rebecca G. Njuguna, Joshua Munywoki, Kara Hanson, Andrew Mulwa, Sassy Molyneux, Isabel Maina, Charles Normand, Julie Jemutai, Edwine Barasa

**Affiliations:** 1 Health Economics Research Unit, KEMRI-Wellcome Trust Research Programme, Nairobi, Kenya; 2 Institute of Healthcare Management, Strathmore University Business School, Strathmore University, Nairobi, Kenya; 3 Health Systems and Research Ethics Department, KEMRI-Wellcome Trust Research Programme, Kilifi, Kenya; 4 Faculty of Public Health and Policy, London School of Hygiene and Tropical Medicine, London, United Kingdom; 5 Directorate of Medical Services, Preventive and Promotive Health, Ministry of Health, Nairobi, Kenya; 6 Centre for Tropical Medicine and Global Health, Nuffield Department of Medicine, University of Oxford, Oxford, United Kingdom; 7 Health Financing Department, Ministry of Health, Nairobi, Kenya; 8 Centre for Health Policy and Management, Trinity College, The University of Dublin, Dublin, Ireland; University of Hong Kong, HONG KONG

## Abstract

Public Finance Management (PFM) practices influence the attainment of health system goals. PFM processes are implemented within the budget cycle which entails the formulation, execution, and monitoring of government budgets. Budget monitoring and accountability actors, structures, and processes are important in improving the efficiency of health systems. This study examined how the budget monitoring and accountability processes influence the efficiency of county health systems in KenyaWe conducted a qualitative case study of four counties in Kenya selected based on their relative technical efficiency. We collected data using in-depth interviews with health and finance stakeholders (n = 70), and document reviews. We analyzed data using a thematic approach, informed by our study conceptual framework. We found that weak budget monitoring and accountability mechanisms compromised county health system efficiency by a) weakening the effective implementation of the budget formulation and execution steps of the budget cycle, b) enabling the misappropriation of public resources, and c) limiting evidence-informed decision-making by weakening feedback that would be provided by effective monitoring and accountability. Devolution meant that accountability actors were closer to implementation actors which promoted timely problem solving and the relevance of solutions. Internal audit practices were supportive and provided useful feedback to health system managers that facilitated improvements in budget formulation and execution. The efficiency of county health systems can be improved by strengthening the budget monitoring and accountability processes. This can be achieved by increasing the population’s budget literacy, supporting participatory budgeting, synchronizing performance and financial accountability, implementing the existent budget monitoring and accountability mechanisms, rewarding efficiency, and sanctioning inefficiency.

## Background

Attainment of Universal Health Coverage (UHC) is a global health priority. Achieving the UHC goal has however been limited by constrained resources in the face of a growing burden of disease [1]. Increasing public funds for UHC can be achieved through either allocating more public, pooled resources to health or by improving efficiency in the use of available resources [2–4]. Increasing government health spending in low and middle-income countries (LMICs) is difficult, with limited economic growth, and competing interests from other government-supported sectors [2]. A potential strategy to increase fiscal space for health is therefore improved efficiency in the use of resources available to the health department [3]. Efficiency entails getting more health from the available resources [5, 6]. There are two types of efficiency, allocative efficiency which is concerned with the best combination of inputs to achieve the maximum possible outcomes/outputs [5] and technical efficiency which entails maximizing outputs/outcomes for a given set of inputs [5].

Enhancing efficiency in health will require addressing inefficiencies in the health sector. Several studies within LMICs have documented challenges with Public Financial Management (PFM) or its components as a source of health system inefficiencies [7–10]. PFM refers to the mechanisms that governments have in place to mobilize, allocate, and expend public resources [5]. PFM happens within the annual budget cycle which entails the formulation, execution, and monitoring, and accountability of the budget [5]. Our previous papers discuss how the budget formulation [11] and execution [12] processes influence health system efficiency in Kenya. This paper focuses on how the budget monitoring and accountability processes influence county health system efficiency in Kenya. Budget monitoring refers to the evaluation of the achievement of budgetary goals. Budget accountability on the other hand entails actors taking responsibility for their budget decisions and the subsequent outcomes[13, 14]. It is important that existent monitoring and accountability enhance rather than deter health system efficiency.

In 2013, Kenya devolved its governance arrangement by establishing a two-tier system with a national government and 47 county governments [15]. Parallel to devolution, Kenya also introduced a new Public Financial Management law (PFM Act 2012) to guide the management of public funds under the new governance arrangement. To explore how health system efficiency within counties in Kenya can be improved, we first conducted an efficiency evaluation of counties using Data Envelopment Analysis (DEA) [16]. The 47 counties in Kenya were allocated an efficiency score between 0–100. We then regressed the efficiency scores against possible determinants of efficiency identified through a systematic review [17] and a stakeholders’ forum [18]. The results of the regression [16], the systematic review, and the stakeholder’s forum identified PFM and its components as determinants of efficiency. In this phase of the study, we sought to explain how PFM processes, specifically budget monitoring and accountability, influence health system efficiency.

## Methods

### Ethics statement

This study received ethics approval from the KEMRI Scientific and Ethics Review Unit (SERU), approval number KEMRI/RES/7/3/1. Informed consent was obtained from all study participants. Approvals from relevant authorities were also sought before the commencement of the study. All methods were performed following the relevant guidelines and regulations. All study participants were provided written informed consent to participate in the study and be audio recorded. Confidentiality was assured by anonymizing the study counties, de-identifying respondent data, securing the collected data in password-protected computers, and restricting access to the data to researchers only

### Conceptual framework

**Fig 1:**
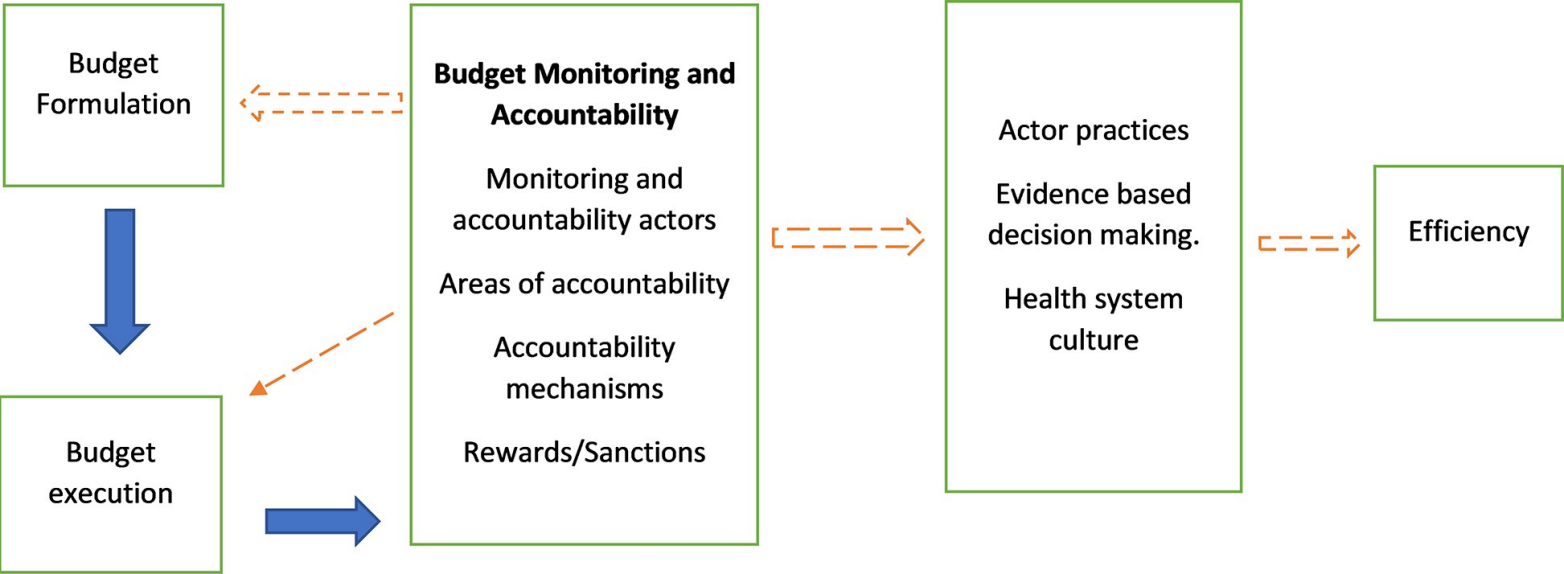
Conceptual framework. Budget monitoring and accountability influenced efficiency by shaping patterns of behavior that either encouraged or discouraged (in)efficiency within the budget cycle.

Through a literature review, we identified four issues around budget monitoring and accountability that influence efficiency (Fig 1): 1) actors responsible for monitoring and accountability actors (internal and external). This involves understanding who bears the responsibility for accountability and to whom they are held accountable. The effectiveness of accountability systems in ensuring health system efficiency is greatly dependent on clarifying the roles of these actors given their interconnectedness [14, 19]. Interpersonal relationships are necessary for creating a supportive environment however they can also be subject to misuse [14, 19]; 2) Monitoring and accountability mechanisms–these pertain to the methods involved in holding these actors accountable. To ensure the effectiveness of accountability systems, well-defined mechanisms must be in place to ascertain the attainment of set goals [20]; 3) areas of accountability–this concerns what exact aspects the actors are being held accountable for. There are three spheres of accountability–financial, political, and performance. Clearly defining the scope of accountability is crucial for defining areas of synergy, contention, and potential gaps [21]. This is important for maximizing the outcomes of the accountability process [22, 23], and, 4) Rewards and sanctions for efficiency and inefficiency. A key aspect of an accountability system is sanctioning unachieved goals, this ensures that the actors are incentivized to make choices that result in efficiency [20]. These four issues influenced efficiency by determining i) Actor practices–whether or not they make choices that enhance efficiency ii) evidence-based decision making–whether the health system can learn from previous decisions) the health system’s culture–the prevailing organizational culture toward efficiency.

### Study design

We conducted a qualitative case study of four counties that were selected to represent two (relatively) inefficient and two (relatively) efficient counties based on efficiency scores identified by the larger Kenya Efficiency Study (KES) [16]. This was based on the hypothesis that more efficient counties would have more effective PFM systems and practices compared to less efficient counties. We further selected the cases to represent other determinants of efficiency identified in quantitative analysis [16] such as the prevalence of HIV and population size (Table 1)

**Table 1:** County profiles.

County	Efficiency Score*	Per Capita County Public Health Expenditure (2018/2019) KES
**A**	0.9	1660.19
**B**	0.9	1580.26
**C**	0.4	2201.33
**D**	0.5	2901.21

### Study cases

Kenya has a devolved system of governance with a national government and 47 sub-national governments, “counties”. The counties are semi-autonomous, with a mandate that includes health service delivery. The counties finance health service delivery using exchequer allocations, their own source revenue collections, and conditional grants. The exchequer allocations form the major source of finances for most counties. Exchequer funds are released as a whole sum. Counties then have the responsibility of allocating the funds between departments, thereafter, planning, executing, and accounting for the resources. Increased accountability and enhanced citizen oversight in the use of public resources was one of the key objectives of devolution [15]. To exercise their mandate of managing resources, counties have a legislature, the county assembly comprising of publicly elected ward representatives, and the executive comprised of appointed heads of departments [15]. The national government allocates funds between the counties and provides oversight and support in the management of these funds.

### Study population and sampling

At the county level, we collected data from the county department of health, and the county treasury and finance. Within the Department of Health, we collected data from the county level, the sub-county level, and health facilities including hospitals and primary healthcare centers. Within the department of finance, we collected data from the administration and the finance and procurement offices, and at the national level from the national treasury and the Ministry of Health(MOH) and development partners. In-depth interviews and document reviews were the main data collection methods used.

### Data collection

#### In-depth Interviews

We purposively sampled participants directly involved in the health budget planning, execution, and accountability processes. We interviewed a total of 70 respondents across the four counties (Table 2). Data collection stopped once theoretic saturation was achieved.

**Table 2:** Respondents sampled.

	County-Level Respondents				National Level Respondents
Interviewee group	A	B	C	D	
Health Managers	4	6	3	3	**1**
Finance Managers	4	1	2	5	**1**
Sub County Health Managers	0	3	2	0	**-**
Facility Health Managers	7	9	5	9	**-**
Donors	-	-	-	-	**6**
Sub totals	15	19	12	17	**8**
Total	**70**				

We used an interview guide developed from the study’s conceptual framework to guide the in-depth interviews. All county-level interviews were conducted face-to-face within the workstation of the respondents. National-level interviews were conducted face-to-face or online depending on the respondent’s preference. All the study participants provided signed informed consent. The interviews were audio-recorded and lasted 45–90 minutes. We conducted in-depth interviews between May and October 2021. Given the nature of the study–qualitative the study participants are identifiable to the study team

#### Document reviews

We collated and analyzed documents with information on budget monitoring and accountability within counties. These included the constitution of Kenya, the Public Finance Management Act, The County Budget Operational Manual, the Controller of Budget Act, the Controller of Budget Regulations, the County Government Act, and the Planning, Budgeting, and Performance Review Process Guide for the Health Sector

### Data analysis

The recordings were transcribed onto Microsoft Word and thereafter transferred to NVIVO for coding and charting, and the data were analysed thematically. Thematic analysis is a method that guides the identification, organization, description, analysis, and reporting of themes found in a data set [24]. We aimed to both compare cases and explore cross-case emerging issues, We immersed ourselves in the data by repeatedly reading through the transcripts. We then developed a thematic framework based on the study’s conceptual framework (Fig 1), while accommodating emerging themes [24].

## Results

The budget monitoring and accountability systems in Kenya are characterized by both formal processes through policies and guidelines, and informal policies brought about by power relationships on the ground. Fig 1 highlights some of the accountability pathways from the community (Community Units and Community) to the health facilities to the sub-county, then county level (county health management team, county executive, county assembly, and the county treasury), and finally the national level (Ministry of Health, The National Assembly, the Senate, the National Treasury, the controller of budget, the auditor general and the national level development partners). In the next sections, we describe budget monitoring and accountability mechanisms on paper within the health system in Kenya. We discuss this using the key aspects of our conceptual framework– 1)who the system is accountable to (monitoring and accountability actors) 2) what the system is accountable for (areas of accountability) 3) How the system is held accountable (mechanisms of accountability 4) sanctions and rewards for accountability. This is followed by the dejure aspects where we discuss findings on the four aspects of budget monitoring and accountability influencing efficiency: accountability actors, accountability areas, accountability mechanisms, and rewards/sanctions for efficiency.

### Budget monitoring and accountability mechanisms on paper in Kenya

There are multiple actors, and requirements for budget monitoring and accountability in Kenya with the overall goal of ensuring the appropriate use of public funds. These processes happen throughout the budget cycle, from budget formulation to execution and the final stage of accountability. We discuss the existent monitoring systems in the Kenyan budget systems as per the conceptual framework.

The county health system is accountable to both the public and to government bureaucracy. Public accountability entails government officers’ answerability to the citizens. This happens in various ways. First is public participation in the planning and budgeting processes, which is a constitutional requirement [15]. After budgets are formulated and before they are approved, they are presented to the public for feedback, this is often done through public hearings as is required by the Constitution and PFM Act. In addition, there are forums such as the stakeholders’ forum and the county business and economic forum (CBEF). These forums bring together county health leaders, county finance leaders, and representatives of various public groups such as associations, the private sector, civil society, and non-governmental organizations. In these sessions, the public through representatives can provide feedback on the budgeting process [25].

Internal accountability for resources allocated to counties happens at both the national and county levels. Table 3 outlines the actors in budget monitoring and accountability processes at the national and county levels in Kenya. At the county level, both the executive and legislative actors provide monitoring of government budgets (Table 3). The executive comprises the governor and the county executive committee members. Their key role in PFM is to implement county legislation including the appropriations bill and to manage and coordinate the departments. The legislative actors in the county are the county assembly. Their mandate includes representation, legislation, and oversight. Specifically for PFM, the county assembly approves the county development plans, and they review and approve the county budgets. When there are accountability challenges related to funds, the county assembly can summon and question the executive and members of the health department. They further have the mandate to impeach any executive member who is misappropriating funds or underperforming [15]. At the national level, the Senate, national treasury, the controller of budget, and the auditor general are key players who are involved in the monitoring and evaluation of county budgets. The national treasury prepares the division of revenue bill and the county revenue allocation bill. The controller of budget on the other hand monitors the execution of budgets by authorizing all withdrawals from county accounts [15]. This is often done monthly, where counties present their expenditure requests to the office of the controller of budget and they have to get approval to incur expenditure. The auditor general conducts audits of counties following the completion of every financial year [15]. This happens annually and it is required 6 months into the next financial year. The Senate approves the county allocation of revenue bill, and it then provides oversight on the use of resources, by for example summoning governors and the county executive to explain any audit queries. Following impeachment by the county assembly, the governors are then to plead their case at the senate which can then validate the impeachment or sisqualify it.

**Table 3:** Budget monitoring and accountability actors within counties in Kenya.

Actor	Role
**Public accountability Actors**	
Public	Public accountability All officers responsible for managing public funds are accountable to the public
**Internal accountability County Level Actors**	
The County Treasury	Primary custodian of county resources. They ensure all county resources are used to promote efficiency.
The Governor	The governor holds the CEC Finance Accountable The governor issues the authority to withdraw county funds
County Executive Committee	They are the executive control within the county and are made up of county executive committee members from various departments
The County Executive Committee Member Finance	They oversee the financial administration of county finance They head the county treasury
County Chief Officers	Chief officers are the Accounting officers of ministries who oversee departmental financial aspects
The County Assembly	The county assembly provides oversight over the county executive committee and the county treasury.
County Assembly Budget and Finance Committee	Monitors all budget matters within counties
**Internal accountability National Level Actors**	
The National Treasury	The chief custodian of Kenya’s financial resources
Office of the controller of budget	Provide oversight over the implementation of the budget Submits statutory reports to parliament on budget implementation.
Office of the Auditor General	Reviews the financial and operational performance of the county

Adopted from the county budget operational manual, the Public Financial Management Act, and the Constitution of Kenya

On paper, the health system is accountable for finances and technical performance. Financial accountability entails tracking and reporting on how public funds are used using accounting tools. In Kenya, this is achieved through financial reports and overseen by the Ministry of Finance, Office of the Controller of Budget (OCOB), and the Auditor General. Performance accountability entails reviewing the attainment of targeted goals. Performance outputs are overseen by the MOH and monitored through performance reviews. For example, perinatal and maternal mortality incidences are reported and audited within 24 hours for maternal mortality and within 7 days for perinatal mortality.

Kenya uses several mechanisms to ensure the public health system is held accountable. These mechanisms include financial audits, financial and performance reports, the requirement for public participation, the requirement for budget transparency, and supervision. Table 4 below summarizes what each of the accountability mechanisms entails.

**Table 4:** Budget monitoring and accountability mechanisms.

Mechanism	Description
Audits	Conducted by the office of the auditor general on an annual basis. The constitution requires that audit reports are availed within 6 months after the completion of every financial year
Budget Implementation Review Reports	Budget implementation review reports are to be made quarterly by the county treasury and sent to the office of the controller of budget. The controller of the budget then consolidates and publishes the report on the controller of budget’s website for the public
Supervision	One-on-one facility supervisions are to be conducted monthly by the county and sub-county health management teams
Annual Performance Review Reports	Done between August and September following the completion of the FY. It entails a detailed review of outputs achieved and it informs the next budget allocation.
Quarterly Public Expenditure Review Reports	Done quarterly, spearheaded by the Department of Health. Reviews both the use of financial resources and performance.
Budget transparency mechanisms	The constitution requires transparency and openness in all budget processes. Most counties publicize their budgets on their websites. These are also publicized by the controller of budget
Participatory budgeting	The constitution of Kenya requires public participation in budgetary decisions. This is achieved through the county budget and economic forum (CBEF) and public hearings. At the CDOH level, this is achieved through County Health Stakeholders Fora and Health Sector Working Group
Budget controls through expenditure approval	Budget controls are applied at all levels of budget execution from the facility level to the county level. Facilities have to be issued with an Authority to Incure Expenditure (AIE) to spend their funds. This is done monthly or quarterly depending on counties Counties present their monthly expenditure plans to the office of the controller of budget for approval before spending

There are several sanctions attached to various aspects of the budget monitoring and accountability process. The county assembly and the senate impose their mandate by sanctioning members who are involved in the misappropriation of public funds and underperformance. The public’s key mechanism for sanctioning is through the electoral process. The office of the controller of budget, on the other hand, may stop counties from spending which may impact the county in totality rather than specific individuals. The other sanctions are centered around the judicial process, which may involve a jail term or the imposition of fines for noncompliance.

### Budget monitoring and accountability mechanism in practice in Kenya

#### Accountability pathways

**Fig 2:**
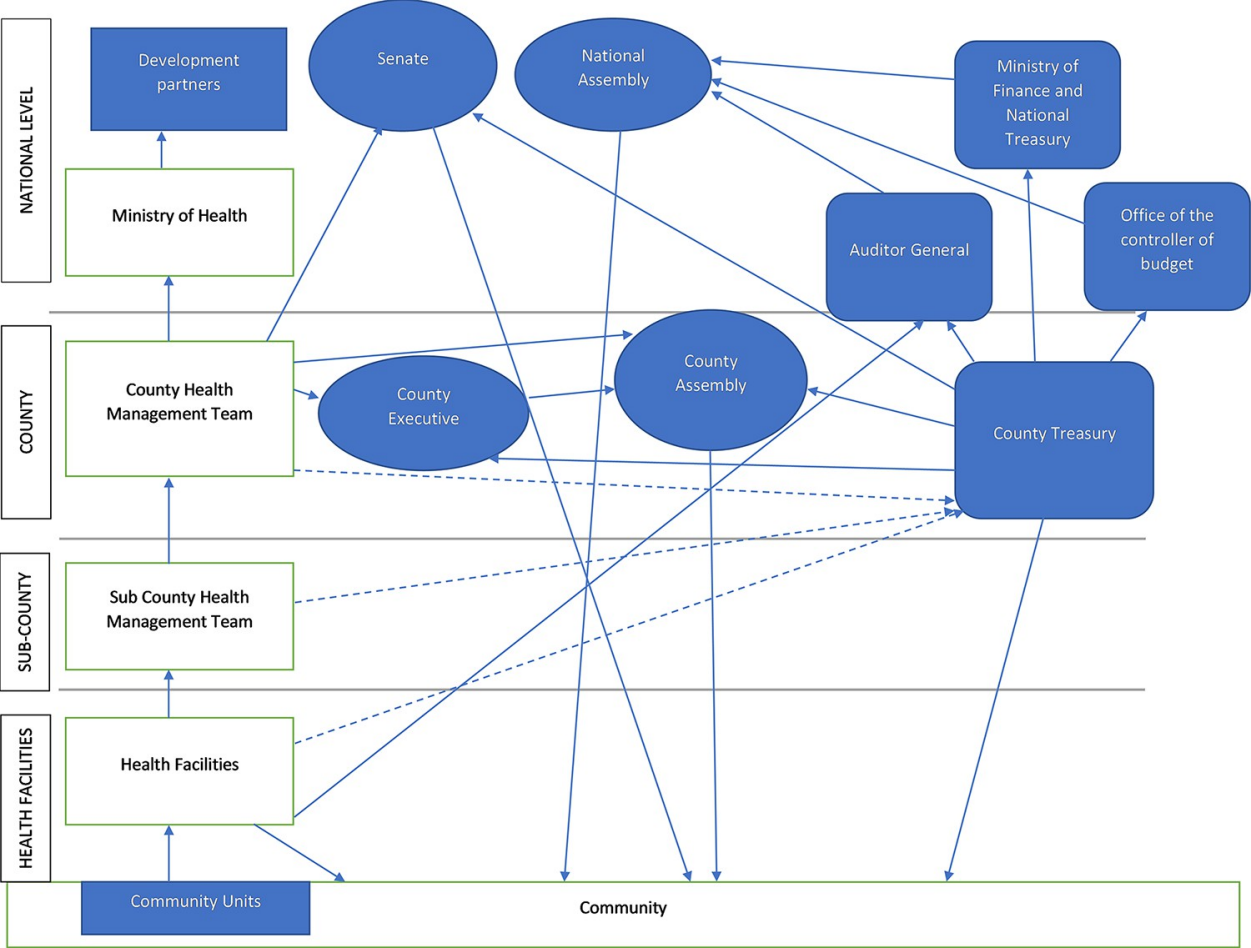
Accountability actors within the county health system in Kenya.

The existent accountability pathways–both internal and public accountability pathways were ineffective in enhancing health system efficiency. The PFM Act 2012 established different accountability mechanisms with an overall goal of enhanced efficiency (Fig 2). These sentiments were also echoed by the respondents who noted two pathways for health system financial accountability that influence efficiency: accountability to the government (“bureaucratic/internal accountability”) and accountability to the public (“external accountability”) (Fig 2). There were challenges in both the internal and public accountability processes that limited efficiency.

### (i) Internal Accountability

#### The personal interests of actors in the internal accountability process conflicted with the health system’s goals of efficiency

The respondents felt that actors tasked with budget monitoring and accountability used their power to front personal goals. This they said compromised health system efficiency. For example, in counties B and C, the county assembly was said to use its oversight power to promote its priorities over the priorities of the health system. Also, in county C, the county health management team was faulted for using their supervisory power to settle scores rather than improve the health system.

“*There’s the oversight role of the county assembly if it is done properly*. *However*, *my experience with this assembly is that it’s not being done properly*. *Sometimes I think it’s even done for the wrong reasons*. *Looking at some of the questions they ask you might say it’s more political than a way of system improvement*.*” Health Facility Manager*, *County C*

#### Internal actors’ conflicting roles within the budget process compromised efficiency

First, the members of the county assembly (MCAs) who were tasked with the role of providing oversight were also involved in budget execution. The MCAs received an allocation from the executive, the ward development fund which they budgeted and executed. The respondents noted that because of their role in execution, the MCAs were unable to hold the executive accountable. The MCAs overlooked the executive’s mistakes to get more allocation to their budget. Consequently, monitoring mechanisms were less effective with possible effects on efficiency.

*“You have MCAs constantly trying to agree with the executive that favors the ward development funds*, *and lets off the executive easily on misappropriations*. *So*, *you hear a lot of gentlemen’s agreements in the counties*. *MCAs are trying to get more money into the ward development funds*. *The governor is trying to get MCAs off their backs…*.*So*, *the net effect is then you have this very weak legislative oversight across the board*.” *National Level Development Partner*

Second, actors tasked with the role of ensuring monitoring and accountability mechanisms were enforced were those to be monitored. Consequently, monitoring mechanisms were ineffective. For example, the county treasuries were said to limit the data required for budget transparency and public participation. The county treasuries who oversaw public participation were also the key implementors of the budget. Hence, they did not welcome scrutiny.

*“Again*, *the people who are tasked to ensure there’s budget openness would rather not have it done*. *So the accounting officers are the ones*, *for example*, *to organize public participation in the budget*, *and they are key beneficiaries of the lack of budget accountability*. *So it becomes difficult for them to do it*” *National Level Development Partner*

#### The proximity of internal actors to the health system enhanced efficiency

County assemblies were reported to correct misperformance in the health system thereby enhancing efficiency. Besides, the presence of oversight power close to the health system meant that challenges were quickly and effectively addressed. For example, some county assemblies helped to lobby for increased resource allocation to the health sector thereby promoting sufficient budget allocation for specific health agendas.

*“The oversight role of the assembly has a two-pronged effect*. *Sometimes the objectives of the county assembly could clash with the health system objectives*. *But there are times that county assemblies have called out the health sector when they are doing certain things in the wrong way*. *There are times the county assemblies have pushed for the change of CEC members for health*, *because of certain omissions that were being done in the health sector*. *There are times that the county assembly members have been able to advocate for increased resources for the health sector for specific priorities*.” *National Level Development Partner*

### (ii) Public accountability

The respondents noted that the existent mechanisms for public accountability (including public participation through local gatherings “barazas”, and incorporation of public representatives into key government committees and decision-making fora) were ineffective in enhancing efficiency. Public participation was limited by 1) the public knowledge and skills to criticize the budget and 2) budget transparency.

*“The constitution has provided for budget public participation but then*, *it has not achieved its objectives of making sure that the public holds*, *the budget holders accountable*. *It has not succeeded*. *As a result*, *we’ve invested in devolution*, *but our return on investment is very low*. *We spend so much money on a small improvement in service delivery*. *I don’t know if there are studies that show*, *for example*, *how much money we use to immunize one child*, *but we are inefficient*. *We are using more than we should to immunize a child given these leakages along the budget process*.” *National Level Development Partner*

#### The public lacked the knowledge and skills to effectively identify and address health system challenges

As a result, the public often blamed the wrong people for health system failures, hence health system challenges remained unresolved. For example, in County B, health workers were blamed for stealing medicine that was not supplied. Medicine was procured and paid for by the county health management and the county treasury with no input from frontline managers. Similarly, in County D, the health system was blamed for poor patient outcomes that resulted from the misappropriation of resources at a higher level.

*“One of the biggest weaknesses with the public is if a patient has a medical complication right now; they will be on social media asking the director of health to account for the condition of the patient*. *However*, *they are ignorant of the fact that somebody who mismanaged funds today is likely to cause the death of a patient one year from now*. *They don’t look at it that way*.” *County Health Manager County D*

*“So I think what is needed is more budget literacy among frontline workers and the public*. *We need more people to understand the budget so that they demand accountability*. *Yeah*, *that is the only way we will improve performance*. *Everyone will know that they are being watched*. *People will know that this budget has been released*, *and this is the evidence for its release*” *National Level Development Partner*

#### The public lacked access to budget data

When those in government failed to provide sufficient information to the public to allow for critique, then public accountability was compromised which in turn compromised efficiency. The constitution of Kenya requires budget transparency to enhance accountability. However, in all four counties, neither the public nor the facility managers in lower-level facilities had full information on the health budget or expenditure. It was therefore difficult to hold the county accountable for resources and outcomes.

*“It’s a challenge*. *When we request budget implementation reports*, *you’ll be asked*, *what use do you have for them*? *This is a very strange question from a government that is required by law to publish and publicize such reports*. *So there are gaps in the level of accountability that government officials have*. *And in some scenarios*, *it leads to this very opaque approach to which governments do their things*. *County XXX is a good example*: *their website is full of photos of the governor launching things*. *But since we started doing our budget transparency survey in 2015*, *XXX has been one of the opaquest county governments when it comes to their budget documents*” *National Level Development Partner*

*“you know controls are made by human beings*. *So I would say the systems we have in the county are not perfect*. *For example*, *you might find that the facility is not getting the recurrent budget*. *And probably the department is getting it*, *but it’s not trickling down*. *Mhm*. *So why is it not trickling down*? *Where is the problem*? *Who is sitting on that money*? *And why are there no follow-ups so that that money gets to where it’s supposed to be*? *So those are the challenges*” *Health Facility Manager*, *County C*

#### Participatory budget and accountability fora such as the Health Sector Working Group, on the other hand, were limited by their functionality. These fora were either partially functional or not functional

Their functionality was either compromised by limited access to funds to run their mandate or limited power over the budget process. For example, the role of hospital boards and committees at the facility level was curtailed by limited provider autonomy. On the other hand at the county level, the role of the Health Sector Working Group (HSWG) was taken up by the treasury. As a result, the health system failed to benefit from both the oversight role and support from these entities.

*“for example*, *the health sector working group is one of the structures that is supposed to help link resources with the results*. *This is because it is comprised of members who are conversant with the health needs of the specific counties*. *They will then have access to information on available resources and high-priority areas*. *But you will find that the health sector working groups are not fully effective in all the counties as we would want them to be*. *And so*, *because of that*, *they sometimes lag or come too late in the day to do their monitoring and evaluation*.” *National Level Development partner*

Second, where these entities existed, they lacked the legitimacy to conduct their mandate. As a result, their recommendations were easily ignored. This further compromised oversight which compromised accountability.

*“So they are there*, *but they are not powerful*. *They have not been given the legitimacy and the power to for their mandate*. *So they may have provisions but those provisions or those*, *if they have*, *if they*, *they have oversight recommendations*, *they can as well be ignored with zero consequences*. *So they’re not powerful*.” *National Level Development Partner*

#### Accountability areas

Participants noted that there were **delinked financial and service delivery performance accountability processes that limited the connection between health system resources and outcomes** thereby compromising efficiency. Delinked accountability for finances and outcomes compromised the process of identification of inefficiencies within the system; it limited the use of data for decision-making and also limited the answerability and responsibility of actors. The delinked accountability mechanisms were a consequence of a systemic separation of financial and technical aspects of health. Financial accountability within the department was spearheaded by the Chief Officer of Health while performance accountability was spearheaded by the Director of Health. Consequently, it was difficult to link resources allocated to indicators achieved. The health system failed to learn from past mistakes/achievements to improve performance.

*“no*, *he is only responsible for accounts*. *For indicators*, *he will only inquire if the funds were used as per the donor’s specifications*. *The sub-county teams*, *on the other hand*, *are the ones who deal with the indicators*. *So*, *if they come for supervision they will ask what’s not happening*. *What are you doing*? *They look at the registers but they never audit funds*” *Health Facility Manager County D*

Respondents also noted that separate financial and service delivery accountability systems compounded by limited provider autonomy limited actors’ responsibility over their (in)actions. In counties B and C, health workers declined to take full responsibility for performance because they lacked financial autonomy. In addition, in all four counties, misperformance was blamed on other entities.

*“shortage of commodities is almost always a daily occurrence*. *Even right now as we speak*, *some commodities are out of stock*. *Patients expect us to provide medicine*, *but they don’t get those commodities*. *We try to explain to them that at times their expectations are beyond our facility because we are not procurement*. *The hospital is not a procuring entity*. *If somebody else hasn’t procured or there is delayed funding or something we might not help as such*.” Health Facility Manager County B

### Accountability mechanisms

The existent accountability mechanisms such as 1) Annual Performance 2) Quarterly budget reviews and 3) Annual financial audit processes.

### (i) Annual Performance Reviews (APR)

#### The respondents noted that APRs were either late, not done, or partially done

The APR process is mainly spearheaded by the MOH at the national level and the director of health at the county level. These actors have limited jurisdiction over financial resources. As a result, the process was either not done or partially done to include the performance data leaving out the financial data. Yet, this was the main process that reviewed targets achieved against set goals and availed resources. This process was to inform the subsequent year’s resource allocations. Because of the failures in the APR processes, inefficiencies were carried forward to subsequent years.

*“If you do the annual performance review properly*, *it will help you to improve the implementation of the next budget*. *For example*, *now we are in the financial year 2021/2022*. *If by the end of August or by mid-September*, *you have already conducted the APR for last year*, *you would already be able to see inefficiencies that you should not carry over*, *to this current year*. *But because of not doing*, *proper evaluations at different stages or doing them late in the day*, *then you carry on inefficiencies*” *National Level Development Partner*

#### The health system lacked the power to implement APR recommendations

The respondents termed this a consequence of limited budget transparency and limited CDOH autonomy over their resources as identified in the budget execution paper. Hence, the health system missed out on key lessons from previous quarters or years that would have improved its performance.

*“When we do it (APR) as health only*, *it’s very difficult for us to understand what happened to the budget*. *Often*, *we do not have answers*. *We just know there was no money*, *why was there no money*? *We do not know*. *If finance was participating and we say we were able to realize sixty percent of the budget then we can discuss the forty percent*. *We can narrow down to the root cause–whether the problem was the department*, *the chief officers*, *or finance*, *that way we can improve subsequent budgets*. *But*, *the way it is done*, *we do not monitor the budget*” *County Health Manager County B*

### (ii) Budget expenditure reviews

#### While quarterly budget reviews were completed on time, they were done to check a box rather than for system improvement

Quarterly budget reviews were a legal requirement that was enforced with repercussions. If county treasuries failed to submit the quarterly reviews, then they would be denied access to funds for subsequent quarters. The county treasury spearheaded the process, and they published the reports in time. However, the health department and the public could not make sense of the data as the figures were aggregated. The aggregation compromised transparency and accountability thereby limiting valuable feedback for improved service delivery.

*“When the county assembly approves the budget*, *they approve the budget so that the nutrition officer can know I have 25 million for my program*. *But when the controller of budget publishes reports*, *they publish aggregated figures*. *They publish quarterly*, *and they have deadlines on how to do that and it’s available online*. *So many counties will hide in the aggregation*.” *National Level Development Partner*

### (iii) Auditing

The respondents noted that the external audit processes were characterized by 1) corruption, 2) lateness 3) Limited feedback, and 4) perverse application, all of which limited their effectiveness as a system for improved efficiency.

#### The respondents noted that the external audit process was marred with corruption

Auditors were said to source for bribes rather than effectively audit to improve the health system. Consequently, the audit process led to limited feedback for efficiency and loss of resources from the health sector. For example, in County B, each department was required by the county executive to contribute KES 2 million each towards paying auditors for a credible audit report. Respondents also noted that the counties that failed to pay bribes would be subjected to negative publicity by auditors with claims of misappropriation.

*“They(auditors) will tell you*, *even before they look at your books*, *do you think we want to go and peruse your dusty books*? *Do you think that is what brought us here*? *We need lunch*. *Use your common sense*. *We are not going to camp here*. *That is what they tell you*. *The person who should have audited you is the one who is asking you to go and withdraw public funds from the bank account* “*Health Facility Manager*, *County C*

The respondents also noted that on some occasions, the audits came too late to influence subsequent budgets. This further limited the effectiveness of feedback received from auditors and the uptake of the recommendations for improved efficiency.

*“There is a program we started implementing in 2017*. *For all those years they never audited us*. *Then*, *they started auditing us in 2021*. *They backdated the audit to 2017*. *Now*, *what would that audit tell*? *If it was timely*, *it would help us know our weak and strong areas so that we can improve in the subsequent years but there is no value in sending an auditor to cover three*, *four years*” *County Finance Manager County D*

#### Respondents noted that feedback following audits was either late or not forthcoming at all

Consequently, the health system missed out on key recommendations for improved efficiency. For example, in County B, respondents noted that they were subjected to interrogations, but it ended there, and no feedback was given. They were therefore unable to put in place any improvement measures from the interrogations. On the other hand, in County D, some reports were so late that the people involved had forgotten all about the audit and therefore could not implement the recommendations.

*“We submit reports*, *we take bank reconciliations*, *they don’t give us reports*. *It Greatly affects performance*, *because you know*, *we are not perfect*.” *Health Facility Manager*, *County C*

#### Respondents noted that audits were used as a tool of intimidation rather than a tool for system improvement

The respondents noted that the county health management used auditors to intimidate managers whom they deemed difficult to handle. For example, in counties B and C, auditors were sent to intimidate facility managers.

*“They will only send auditors because they are targeting you*. *They want to punish you…Auditors in this county are sent with an ill motive*. *They are sent mainly to discipline managers*, *it’s not objective at all*. *There’s no objectivity in sending the auditors*” *Health Facility Manager County C*

#### Occasionally, internal audits provided an opportunity to identify health system challenges and to give feedback to managers

This enabled the health system to identify and correct inefficiencies. For example, in County D auditors visited facilities to find out the facility challenges so that they could intervene. In addition, the county noted that through audit reports, they have been able to improve their financial performance over the years.

*“We are health workers we are not procurement or finance officers*. *So often*, *we commit one*, *two*, *or three offenses*…*knowingly or unknowingly*. *For example*, *the finance person will tell you*, *that you exceeded your expenditure on item X*. *But you will tell him this mother needed this*. *Or I needed this for my facility to keep on running*. *But we meet at the midpoint and they understand our circumstances*.”*Health Facility Manager County D*

*“Yes*. *In fact*, *for us the county of XXXX*…*we have been able to perform better*…*in the subsequent financial years*. *We*, *started with a disclaimer and*, *uh*, *we endeavored to perform better*. *And at least*, *over the last years*, *I can say we have been able to get a favorable audit opinion*. *That shows*, *improvement*. *Previously*, *we were performing quite badly*. *So it has helped us to improve on the gaps*” *County Finance Manager County D*

#### The reward system for efficiency

The study found the reward system to influence efficiency through 1) the existence and enforcement of rewards and 2) whether the sanctions were deemed punitive and the rewards a motivator.

#### The respondents noted that there were limited deterrents for inefficiency or rewards for efficiency

Good performers were unrewarded and sometimes victimized while poor performers would get away with inefficiency. Where sanctions existed, these were rarely implemented. This encouraged the misappropriation of health system resources and non-performance.

*“Who will punish them [for inefficiency]*, *and we have the bigger fish there*, *who are behind it*? *Who will punish them*?” *County Health Manager County B*

*“Definitely*, *yeah*, *if I can misuse money this year*, *next year*, *and the other year*, *and there is nothing that comes out of it in terms of accountability or punishment*, *the person who comes after me*, *will do the same*. *Yeah*, *so definitely*, *that means inefficiency in terms of converting finances to service delivery*.” *National Level Development Partner*

Respondents also noted that the citizens failed to encourage efficiency by electing leaders who failed to deliver. Service delivery did not always influence the voter’s choice of a leader.

*“as it stands now*, *the public also does not connect low performance with bad budget performance*. *So service delivery does not translate to voting influence*. *So empowering the public person also may*, *influence service delivery*, *because the governor now is aware that if he underperforms then he may as well not be voted in*. *But of course*, *that is a long way coming given the peculiar ways that Kenyans choose their leaders*.” *National Level Development Partner*

It was further noted that in isolated circumstances where sanctions were applied, the offenders found ways to avoid punishment. This limited the effectiveness of the sanctions. For example, in County C, the county assembly impeached the CEC health for underperformance, however, the CEC used the judicial system to evade the impeachment.

#### Respondents noted that perversely applied sanctions limited efficiency

This happened when managers rushed to impose sanctions without proper investigations. This demotivated health workers thereby lowering their performance. For example, in County B, facility managers were sanctioned for using facility revenue at source to avert healthcare crises.

*“if your boss is not supportive and he is in a hurry to sanction you for trying to solve a situation it does not auger well with the other staff who are also in the same scenario because I know most of the med sups are in the same scenario*. *They find themselves in a situation whereby there is nothing they can do*, *services have to continue*, *and they don’t have the finances*. *So*, *when they see their colleague being sanctioned for trying to solve health system challenges*, *there is a tendency for people not willing to take up positions like med sups*. *Besides*, *people will fear spending at the source in the name of they will be victimized*. *But when they don’t spend the service is not offered so you are in a fix*. *You can’t help solve a problem because you are fearing*, *the only alternative that you have*, *that you can use is going to cost you as an individual a problem*” *County Health Manager County B*

#### Respondents noted that sanctions that were not punitive encouraged inefficiency

The offenders did not consider the sanctions harsh enough to discourage bad behavior such as misappropriation of public funds. For example, in counties, B and D health managers transferred when they misappropriated public resources. The respondents noted that this sanction did not match the gravity of the mistake therefore it was not a deterrent for inefficiency.

*“If funds are misappropriated*, *yes*, *I would say*, *but ah the actions I’ve seen taken so far just transfer from one station to the next*. *You know*, *this normally happens*, *because we don’t have what we call performance contracts for public offices in this county*. *If we had performance contracts*, *then that feedback system program would be effective in ensuring that those who perform are rewarded*, *and those who don’t perform are probably demoted or taken somewhere else*. *Yeah*. *Yeah*” *Health Facility Manager County B*

#### Case comparisons

Table 5 presents a summary of the findings for each of the case study counties. Overall we found that there were no systematic differences in findings between the relatively efficient and relatively inefficient counties.

**Table 5:** Results summary.

County/Issue	County A	County B	County C	County D
internal accountability structures	County-level monitoring was fairly Effective	Not effective	Not effective	Not effective
Public accountability	Fairly effective especially at the facility level	Not effective	Not effective	Fairly effective at the facility level (Health Facility Boards)
Accountability Areas	Parallel financial and technical performance reports	Parallel financial and technical performance reports	Parallel financial and technical performance reports	Parallel financial and technical performance reports
Accountability Mechanisms -APR/ Budget Reviews/ Audit	Not comprehensive does not include deliberation with all actors / timely feedback	Not comprehensive does not include deliberation with all actors / timely feedback	Not comprehensive does not include deliberation with all actors /timely feedback	Not comprehensive does not include deliberation with all actors /timely feedback
Punishments/ Rewards	County-level sanctions for misappropriation of funds that were deterrents such as salary deductions Rewards for good performance	County-level sanctions that were not deterrents such as transfers	No sanctions for inefficiency nor rewards for efficiency	County-level sanctions that were not deterrents such as transfers

## Discussion

This study examined the budget monitoring and accountability processes within county health systems in Kenya. We found that the existent budget monitoring and accountability processes had several challenges. First, actors involved in monitoring and accountability had conflicts of interest, were corrupt, demotivated, or were not equipped to perform their roles. Second, there were parallel accountability structures for finances and performance. Third, while there were several mechanisms for budget monitoring and accountability, these were either partially implemented or not implemented at all. Finally, there were no incentives for efficiency nor disincentives for inefficiency. The challenges with the budget monitoring and accountability processes influenced efficiency in various ways.

First, weak monitoring and accountability mechanisms potentially reduce the effectiveness of the budget formulation and implementation processes of the budgeting cycle which in turn affects the efficiency of county health systems. Our previous work has demonstrated aspects of budget formulation that influence health system efficiency including, budget ceilings, the budget structure, participatory budget formulation, the pooling and allocation of resources, and budget approval processes [11]. We also found that the following aspects of budget execution may influence health system efficiency–budget credibility, cash disbursement processes, procurement processes, provider autonomy, and financial management information systems [12]. When these aspects of the budget cycles are inadequately monitored and held accountable, the efficiency of county health systems is likely compromised.

Second, weak monitoring and accountability mechanisms compounded by sanctions that were not deterrents encouraged the misappropriation of public resources during budget execution. Auditors paid to ‘look away’ took away resources from the health system leading to wastage of health system resources. Similar findings were reported in South Africa where failure to impose sanctions for misuse of public funds led to years of non-performance and bad audit reports [26, 27]. In Kenya, this is compounded by the conflict of interest among actors who are responsible for budget monitoring. When actors were tasked with both implementation and ensuring monitoring and accountability, they were unlikely to deliver on their role of budget monitoring and accountability. The actors would limit budget transparency thereby frustrating efforts toward public accountability. That notwithstanding, actors would protect their cronies from facing sanctions for misappropriation thereby encouraging inefficiencies. In Thailand, enhanced public accountability, through active social media handles, was shown to improve health system efficiency [28]. In South Africa, because of conflicts of interest, most health workers were protected from sanctions [26].

Third, budget monitoring and accountability processes influenced the use of evidence-based decision-making. Monitoring and accountability processes failed to provide feedback to enable improved efficiency in budgeting in subsequent years. Failure or incomplete monitoring limited the data available for decision-making. Lack of feedback from accountability processes such as audits denied the health system important information to inform budget decisions. Budget decisions that were not evidence-informed influenced health system efficiency by allocating resources with limited consideration for health system needs and priorities [11]. Besides, monitoring and accountability processes and actors created patterns of behavior for (in)efficiency. The budget is a cyclic process, that is dependent on feedback from previous years to inform future choices and decisions. A culture of misperformance or misappropriation creates a pattern of behavior that encourages inefficiency. Similar patterns that led to years of inefficiency were reported in South Africa where inefficient practices were carried forward for 12 years despite audit reports. These patterns of interactions were finally resolved by extensive sanctions across the health system [26].

Fourth, budget monitoring and accountability processes influenced the health system culture toward efficiency. Because of the dysfunctional monitoring and accountability mechanisms, there were limited efforts to achieve the health system’s goal of efficiency. On the one hand, ineffective annual performance reviews, and budget implementation reviews limited the evaluation of the budget as a means to achieve health outcomes. On the other hand, parallel performance and financial accountability processes limited the linkage of resources with performance. In addition, it created accountability loopholes as it was difficult for health workers to take ownership of their performance when they had no control over their resources. Similar findings have been reported in Tanzania and South Africa where health workers, who lacked control over resources, refused to be responsible for poor outcomes that were linked to the budget process thereby limiting efficiency [7, 29]. Also in Ghana, parallel structures limited the synergistic efforts required to move toward efficiency [30].

Besides the challenges, we found some positive practices around monitoring and accountability that enhanced health system efficiency. First, having accountability actors close to the ground meant that health system issues were resolved quickly, and the proposed solutions were more relevant to affected communities. These sentiments have also been shown by a systematic review of 25 countries, where having power “close to the ground” led to flexibility in resource allocation and more relevant decision-making that was linked to efficiency [31].

Second, internal audit practices were said to be friendly and provided positive feedback to the health system. Besides, it provided an opportunity where facility managers would directly engage with county managers and share challenges, experiences, and proposed solutions. Punitive audits and financial management systems in South Africa were shown to create compliance out of fear and poor working relationships between finance and clinical teams which can likely compromise performance [29].

This study sought to compare monitoring and accountability practices in efficient and inefficient counties. The study did not find any systematic differences in the budget monitoring and accountability practices across the efficient and inefficient counties. This could be because the challenges with the budget monitoring and accountability practices documented are perverse in Kenyan counties, with differences in degrees across countries that are difficult to tease out using a qualitative approach. It could also be because the counties that were ranked as efficient by the quantitative analysis by being on the efficiency frontier are inefficient in absolute terms, even though they are relatively more efficient than the counties that are at a distance from the efficiency frontier.

This study had several limitations. First, the selection of only four out of the 47 counties in Kenya may limit the generalizability of the results. However, since the budget monitoring and accountability processes are similar across counties, the study findings may provide useful insights to other counties. Second, the study did not collect data from patients or civil society who would have provided useful insights, especially on public accountability. The limitations notwithstanding, the study provides potential policy levers for improved efficiency through improved budget monitoring and accountability processes. First, the government should have demarcated roles for implementing and monitoring actors. In addition, the government should empower all the oversight actors with the capacity and resources to perform their roles. Second, the government should provide civic education to the public on their role in public accountability and institutionalize public participation in the budget evaluation as is the case in budget formulation [32, 33]. Third, the health stakeholders’ fora, working groups, and committees should be fully operationalized. Fourth, the national and county governments should encourage the use of synchronous accountability mechanisms such as annual performance reviews for financial and performance monitoring. The health system should be held to account not just for adhering to procurement guidelines, but also for the outcomes that result from invested resources. Fifth, the national and county governments should fully implement and provide feedback following budget monitoring and accountability mechanisms such as annual performance reviews, budget implementation reviews, participatory budgeting, and auditing. Sixth, the national and county governments should impose existent sanctions for inefficiency and implement and enforce rewards for efficiency. However, punitive sanctions should go hand in hand with supportive regulation which is equally effective in enhancing efficiency. Seventh, the county and national governments should increase budget transparency, including access to detailed budget implementation reports. Transparency should cut across the system, including internally to primary care centers and externally to the public. Finally, programme-based budgets provide a mechanism for budgeting that is linked to outcomes. The full implementation of the PBBs will likely enhance efficiency.

## Conclusion

This study has identified budget monitoring and execution issues within county health systems in Kenya that have implications for the inputs, outputs, outcomes, and ultimately the efficiency of the health systems. These implications are mediated through impacts on actor practices, evidence-based decision-making, and inefficient and efficient practices. Given the cyclic nature of the budget process, the interactions of the actors and laws in the budget monitoring and accountability processes lead to patterns of behavior that influence subsequent budget formulation and execution practices. Further research is needed on how public participation can be better enhanced for improved health system efficiency.
